# Evaluation of atmospheric-plasma-source absorption mode Fourier transform Orbitrap mass spectrometry for chlorinated paraffin mixtures

**DOI:** 10.1007/s00216-024-05450-2

**Published:** 2024-08-13

**Authors:** Claudia Masucci, Konstantin O. Nagornov, Anton N. Kozhinov, Kevin Kraft, Yury O. Tsybin, Davide Bleiner

**Affiliations:** 1 Swiss Federal Laboratories for Materials Science and Technology, Überlandstrasse 129, 8600 Dübendorf, Switzerland; 2 Department of Chemistry, University of Zürich, Winterthurerstrasse 190, 8057 Zurich, Switzerland; 3 Spectroswiss, 1015 Lausanne, Switzerland

**Keywords:** Plasma, FTMS, Mass spectrometry, FTMS Booster, Resolution, LS-APGD

## Abstract

Chlorinated paraffins (CP) are complex molecular mixtures occurring in a wide range of isomers and homologs of environmental hazards, whose analytical complexity demand advanced mass spectrometry (MS) methods for their characterization. The reported formation of chlorinated olefins (COs) and other transformation products during CP biotransformation and degradation can alter the MS analysis, increasing the high resolution required to distinguish CPs from their degradation products. An advanced setup hyphenating a plasma ionization source and an external high-performance data acquisition and processing system to the legacy hybrid LTQ Orbitrap XL mass spectrometer is reported. First, the study demonstrated the versatility of a liquid sampling atmospheric pressure glow discharge, as a soft ionization technique, for CP analysis. Second, enhanced resolution and sensitivity provided by the absorption mode Fourier transform spectral representation on this legacy mass spectrometer are shown. The developed Orbitrap-based platform allowed the detection of new isotopic clusters and CPs and COs to be distinguished at medium resolution (setting 30,000 at *m*/*z* 400, ~ 400 ms transients), and even chlorinated di-olefins (CdiOs) at higher resolution (setting 100,000 at *m*/*z* 400, ~ 1500 ms transients). Overall, such proof-of-principle instrumental improvements are promising for environmental and analytical research in the field of CP analysis.

**Graphical Abstract:**

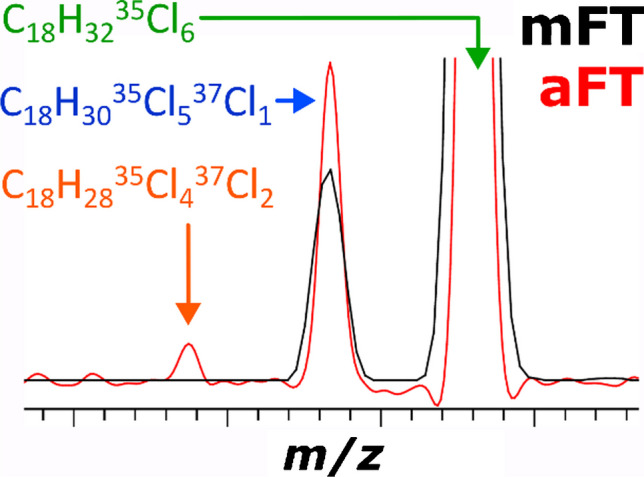

**Supplementary Information:**

The online version contains supplementary material available at 10.1007/s00216-024-05450-2.

## Introduction

Chlorinated paraffins (CP) are a vast group of synthetic compounds widely employed in industrial applications, such as lubricants, plasticizers, and flame-retardants, to name a few [1]. More than one million tons of CP are produced annually by radical chlorination of the respective n-alkanes [2]. The CP have a general molecular formula (C_n_H_2n+2-x_Cl_x_) characterized by a wide range of carbon chain lengths and different chlorination degrees. Overall, CP consist of various C homologs and Cl homologs (Cl_2-20_), making the samples complex mixtures [1, 3–5]. CP have been detected in various environmental matrices such as air, water, soil, and sediment, as well as in biota, including fish, birds, and marine mammals [6–14]. Short-chain CP (C_10-13_) are classified as toxic [15].

State-of-the-art analysis of CP employs mass spectrometry (MS) coupled with gas chromatography (GC) via electron-capture negative ionization (ECNI) or liquid chromatography (LC) coupled with soft ionization techniques such as electrospray or atmospheric pressure chemical ionization (ESI and APCI) [15–19]. Ayala-Cabrera et al. have applied atmospheric pressure photo-ionization (APPI) in a combination with GC–MS to determine and quantify short-chain CP in fish samples, utilizing the selective formation of [M + Cl]^−^ ions and an internal normalization [20]. Nevertheless, quantification remains a major challenge, if all the species are not known (qualitative information) and reference standard materials are not available.

In recent years, a simplification trend toward MS analysis with a direct sample injection (infusion) has been observed [21]. Nevertheless, the complex nature of CP mixtures presents an analytical obstacle [7]. Difficulties are prominent when different instrument types or quantification methods are utilized. For instance, ECNI, which is the predominant ionization technique employed for CP analysis, can be influenced by co-eluting compounds or degradation products. The intense fragmentation observed in electron ionization (EI)-MS(/MS) limits its utility to generic CP analysis, lacking the capacity to differentiate CP groups or homologs. In addition, MS may overestimate the degree of chlorination in higher chlorinated CP due to an increased ionization efficiency [10, 22, 23]. Furthermore, CP are usually found together with their transformation products, such as chlorinated olefins (COs) and chlorinated di-olefins (CdiOs), strongly interfering with their detection. The high-resolution MS (HRMS) is needed to resolve the isotopic distributions of CP and CO compounds that overlap due to the minor mass differences between the chlorine and carbon isotopes. For example, the monoisotopic peak of a CP compound C_18_H_32_Cl_6_ and the second isotopologue of a CO compound C_18_H_30_Cl_6_ differ by ~ 18 mDa at *m*/*z* 458 (if detected as radical anions) requiring resolution exceeding 40,000 to baseline resolve these two peaks.

To overcome these issues, there have been advancements in ionization sources as well as data capturing. For the former, cold plasma-based sources are emerging as soft, flexible, and sensitive solutions which require thorough investigation to understand the underlying chemistry. Notably, the liquid sampling atmospheric pressure glow discharge (LS-APGD) ionization stands out as an innovative technique for combined atomic and molecular analysis [24, 25]. A liquid sample is directly introduced into a micro-discharge; i.e., a low-pressure gas undergoes ionization to form a plasma, which ionizes the analytes in the sample microdroplets. The complete droplet consumption makes it ideal for quantitation. Its rapidity makes it efficient for various applications without requiring extensive sample preparation. LS-APGD has been proven capable of ionizing analytes from elemental species to low-polarity polycyclic aromatic hydrocarbon compounds [8, 26]. However, the formation of adduct or fragment ions during ionization could complicate the interpretation of mass spectra with insufficient resolution, leading to potential false identifications or difficulty distinguishing closely related compounds. These types of challenges can be overcome by HRMS that offers enhanced selectivity and improves the identification and quantification of specific analytes in complex matrices. The HRMS performance can be provided by Orbitrap Fourier transform mass spectrometry (FTMS) with absorption mode FT strategies [27–29].

In FTMS, both the ion’s identity and quantity are encoded within time-domain signals, termed “transients” [30]. Mass spectra emerge from Fourier transformation (FT) of the transients, by suitable calibration strategies. Contemporary FTMS resorts on three alternative modes for signal processing [30, 31]:

- magnitude FT (mFT), in which the amplitude modulus (absolute value of a complex number) distributions are captured and for which an incoherent superposition summation is done. The mFT spectral representation suffers from limitations in resolution and sensitivity (mass spectra contain only positive data points, limiting the mean of noise value upon spectral averaging).
- absorption FT (aFT), in which the amplitude and phase distributions are captured, as a coherent summation. The aFT spectral representation is data intensive and prone to artifacts, e.g. when the ion initial phase is not interscan stable, but offers the benefits of higher resolution (doubling the mFT performance) and sensitivity (both negative and positive data points are present in mass spectra, offering a mean of noise close to zero upon spectral averaging).
- enhanced FT (eFT), which is a more recent compromise between the former two: the aFT peak resolution and mFT baseline (only positive data points) [31].

The eFT mass spectra were introduced exclusively for the Orbitrap models, starting with the LTQ Orbitrap Elite™ [32]. Predecessor models, including the LTQ Orbitrap XL™ [33], which is the platform of this study, were restricted to producing mFT mass spectra. Thus, equipping the LTQ Orbitrap XL with the aFT mass spectra representation capability is desirable. Notably, the data are contained in the aFT mass spectra mirrors found in the transients. Conversely, the information content in mFT and eFT mass spectra experiences reduction when compared to aFT.

Recent developments have shown that, when provided with transients for post-processing, it is possible to generate aFT mass spectra by phasing the ion signals for two primary FTMS instruments: Orbitraps and ion cyclotron resonance (ICR) [34–37]. These software-driven approaches have been enhanced and streamlined by introducing in-hardware phased transient acquisition using external high-performance data acquisition and processing (DAQ/P) systems [29, 38, 39]. Utilizing these systems for transient acquisition offers several benefits over the traditional method of transient detection and processing in FTMS, as described for different Orbitrap and ICR models [40–44]. However, achieving the desired aFT performance with the mFT-only LTQ Orbitrap XL instrument may appear unrealistic due to the anticipated variations in the initial ion phases and potential inaccuracies in triggering signals in this generation of Orbitrap instruments.

Here, we detail the first successful integration of an external high-performance DAQ/P system with the continuous beam ion source LTQ Orbitrap XL, facilitating access to the aFT mass spectra. The enhancement, in conjunction with the LS-APGD ion source, elevates this Orbitrap’s analytical performance, addressing the inherent challenges of CP analysis.

## Experimental methods

### Materials and chemicals

Solvent and blank were HPLC-grade methanol (Sigma-Aldrich, Schnelldorf, Germany) and Milli-Q water (conductivity 0.056 µS cm^*−*1^, Millipore Milli-Q, Serv-A-Pure, Bay City, USA) in a 70:30 mixture. To calibrate the instrument and investigate the mass accuracy of the set-up, Pierce™ LTQ Velos ESI positive and negative ion calibration solutions (product numbers, respectively: 88323, 88324) from Thermo Fisher Scientific (Langerwehe, Germany) were used. The calibration mixture contained caffeine (200 µg), MRFA (10 µg), and ultramark 1621 (0.001% v/v) in a solution of acetonitrile/methanol/water/acetic acid (50/25/24/1) per 10 mL of solution. The single-chained C_18_ CP were synthesized as described previously [23, 45]. The CP were stored in a 500 ng/µL stock concentration in a methanol–water (1:1) mixture. Diluted samples were generated from the stock solutions ranging from 1 to 8 ng/µL.

### Plasma ion source

The licensed (Pacific Northwest National Laboratory, Richland, WA, USA) LS-APGD ion source was described elsewhere [26, 46]. The LS-APGD was mounted in place of the original ESI source via a custom interface. The LS-APGD configuration features a solution electrode that acts as the cathode, and a 90° positioned stainless-steel counter electrode serving as the anode with a positive potential. A microplasma is lit on the liquid surface, ionizing the sample molecules, with the resultant ions channeled into the MS via the ion transfer capillary. Analyte solutions were directly introduced into the LS-APGD microplasma using a 5-mL Luer Lock syringe (Fusion 100, Chemyx, Stafford, TX, USA). A sheath gas flow of He (99.99%, Pangas, Switzerland) is introduced through the stainless-steel capillary of the anode. The solution ground cathode that delivers the analyte is made out of an outer hollow stainless-steel capillary (316 SS, 1.6 mm outer diameter, 0.8 mm inner diameter, McMaster-Carr, Elmhurst, IL, USA) and an inner capillary made of fused silica (inner diameter 250 µm, outer diameter 360 µm, Molex, Lisle, IL, USA). Experimental parameters of the LS-APGD, such as gas flow rate, liquid flow rate, and discharge current, are controlled using a custom-made control box (GAA Custom Electronics, Kennewick, WA, USA). The following LS-APGD conditions were used for all experiments in this work: helium sheath gas flow rate was 500 mL/min, liquid flow rate with the analyte 30 µL/min, discharge current 30 mA (0–1 kV, 10 kΩ), sampling distance 1.0 mm, and an electrode gap of 0.8 mm.

### Mass spectrometer

The mass spectra were acquired using an LTQ Orbitrap XL mass spectrometer (Thermo Fisher Scientific, Bremen, Germany) hyphenated with an LS-APGD ion source. The LTQ Orbitrap XL instrument was calibrated in both positive and negative ion modes using Pierce™ LTQ Velos ESI calibration solutions using the conventional ESI ion source. The LS-APGD Orbitrap experiments were run in the negative polarity to analyze the CP in a mass range *m*/*z* 200–1500 at the resolution settings 30,000 and 100,000 at *m*/*z* 400 (separate experiments). The corresponding transient lengths are 384 ms and 1536 ms (mFT spectral representation). The automatic gain control (AGC) target was set to 10^4^ or 10^6^. The ion injection (accumulation) times were optimized regarding the AGC target values. The investigated injection times were 100 ms, 250 ms, 500 ms, 750 ms, and 1000 ms. The Orbitrap was operated via a standard in-built DAQ system and instrument control software (Xcalibur, Thermo Fisher Scientific).

### External data acquisition and processing system

The LTQ Orbitrap XL instrument was externally interfaced with a high-performance data acquisition and processing (DAQ/P) system (FTMS Booster X2, Spectroswiss, Lausanne, Switzerland), Figure S1, Supporting Information. Previously, similar DAQ/P systems were interfaced with the more recent Q Exactive Orbitraps [29, 38, 43]. There are principal differences in the construction of the prior generation, LTQ Orbitrap XL, and the newer generation, Q Exactive, instruments. The prior generation instruments were designed to exclusively deliver the mFT mass spectra. In addition, the initial period in their transient subject to perturbations in the switching of the high-voltage electronics was substantially longer than in the newer generation instruments—on the order of ms. The latter is a disadvantage for properly phasing the ion signals in the transients as the point of phase coherence for all ions in the mass analyzer is thus further away. Finally, the quality of the triggering signals (e.g., trigger signal rise time and timing) in the prior generation Orbitraps is not as accurate as in the newer generation instruments. These triggering signals are input to the DAQ/P system, at the time ions are ejected from the C-trap, on their way to the Orbitrap mass analyzer [47].

It is thus logical that the generation of the in-hardware phased transients and aFT mass spectra was first reported for the newer Orbitraps. However, in this work, we have successfully addressed the problems of the prior generation Orbitraps and enabled the generation of the in-hardware phased transients and the corresponding aFT mass spectra. To do so, we employed two triggering signals to form sufficient quality start and stop triggers, instead of a single triggering signal that is sufficient for the newer generation Orbitraps. The firmware of the FTMS Booster X2, including the Xilinx and LabVIEW programming of the on-board field-programmable gate array (FPGA) chip for the in-line digital signal processing, has been developed to match the signal quality and triggering logic of the LTQ Orbitrap XL.

Ion signals generated with the induced ion current detection system of the Orbitrap mass spectrometer are first differentially amplified with the original pre-amplifier located near the mass analyzer (Figure S1). After the pre-amplifier, the ion signals as analog transients are sampled (digitized) with a conventional original manufacturer’s in-built DAQ system and yield mass spectra in the magnitude mode FT (.RAW mass spectra). In parallel, a minor part of the analog ion signals is taken after the pre-amplifier and sent to the external high-performance DAQ/P system (FTMS Booster X2). As the time-domain ion signals enter the DAQ/P system, they are first amplified with the user-defined amplification gains using the custom-built amplifier (Spectroswiss). The amplified ion signals are digitized with a digitization (sampling) frequency of around 250 MHz on a 14-bit digitizer (National Instruments, Austin, TX, USA). The digitized ion signals are first processed with the real-time digital signal processing on the embedded FPGA chip. Then, the pre-processed data is rapidly transferred by the high-speed chassis (National Instruments) to be further processed on the central processing unit (CPU) of the onboard controller (National Instruments).

The transients, recorded with the FTMS Booster X2, were converted into aFT mass spectra using Peak-by-Peak Base Edition software (version 2023.4.1, Spectroswiss). In addition, FTMS data, including transients, isotopic envelopes, and broadband mass spectra, for the organic compounds of interest, were simulated using a dedicated software tool, FTMS Simulator (Spectroswiss) [30]. The latter tool was used to perform in silico transient generation for the parameters specific to ion detection with an LTQ Orbitrap XL instrument.

## Results and discussion

### Experimental set-up characterization: calibration mixture analysis

**Fig. 1:**
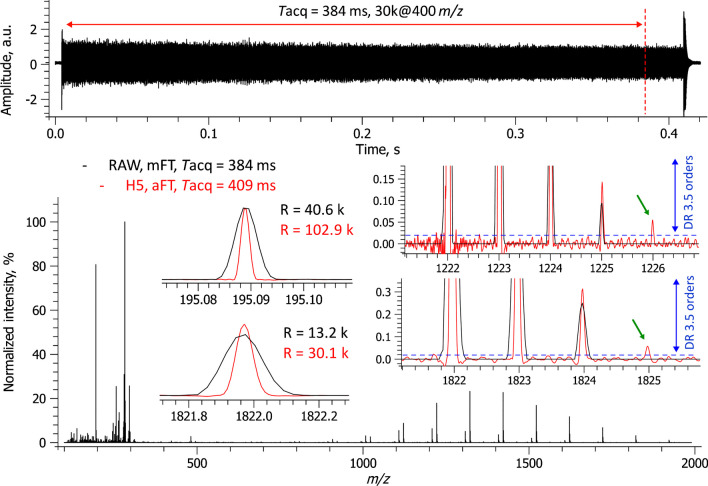
Calibration mixture analysis in a positive ion mode with an LTQ Orbitrap XL equipped with a glow discharge ion source and a high-performance DAQ/P system. A single-scan mass spectrum is shown (positive ion polarity, resolution setting 30,000 at *m*/*z* 400, AGC 2e6, ITmax 200 ms). The resolution increase between the mFT (.RAW, reduced profile) and aFT (.H5, full profile) mass spectra is as expected and confirms the high performance of the external DAQ/P system. An excellent spectral dynamic range of 3.5 orders of magnitude is shown for a single scan

The experimental set-up comprises three primary components, interfaced together for the first time: (i) an LS-APGD ion source, (ii) an LTQ Orbitrap XL mass spectrometer, and (iii) a high-performance DAQ/P system (Figure S1). The experimental set-up underwent an initial characterization and assessment using a standard calibration mixture widely employed in Orbitrap FTMS. The LS-APGD ion source efficiently produced protonated species of the calibrants across a broad mass range. A single-scan mass spectrum of this mixture, performed in positive ion mode, displayed the expected ion signals from caffeine, the MRFA peptide, and ultramark (Fig. 1).

Additionally, the data show that a 409-ms transient signal was successfully recorded using the external high-performance DAQ/P system. This duration slightly surpasses the anticipated transient length of 384 ms, based on a resolving power setting of *R* = 30,000 at *m*/*z* 400, by a 25-ms overhead. Notably, the 409-ms transient period was not directly selected by the user, but is a result of a user-selected resolution setting that required a 384-ms transient and the allied Orbitrap system overhead of 25 ms. Using the FTMS Booster X2 system, a single-scan transient enabled the direct generation of an absorption FT (aFT) mass spectrum employing half-window apodization, with no discernible peak shape anomalies (Fig. 1, insets). As expected, the aFT peak resolution doubles that of the initial mFT spectral output. A single-scan mass spectrum reveals a remarkable spectral dynamic range (DR) spanning 3.5 orders of magnitude. Furthermore, some low-abundance peaks appear solely in the aFT mass spectrum and are absent in the mFT data (Fig. 1, insets). This difference might be attributed to the characteristics of the DAQ systems used, rather than a diminished profile representation in the mFT mass spectrum, considering that it is based on single-scan data. Therefore, the external high-performance DAQ/P system has showcased its potential to capture in-hardware phased transients, even with the initial generation LTQ Orbitrap instruments.

In FTMS, averaging multiple transients or mass spectra is crucial for enhancing data statistics [29]. The projected analysis of CP-containing samples is performed via direct infusion using the LS-APGD ion source and requires averaging numerous single-scan mass spectra or transients. Such data averaging can be vulnerable to scan-to-scan frequency shifts, leading to cumulative data artifacts, which may manifest as a drop in resolution or split peaks. The employed experimental configuration demonstrates remarkable interscan frequency stability, as is evidenced by the ability to average multiple (100) transients without compromising resolution or peak shape quality, as depicted in the aFT mass spectrum (Figure S2, Supporting Information).

**Fig. 2:**
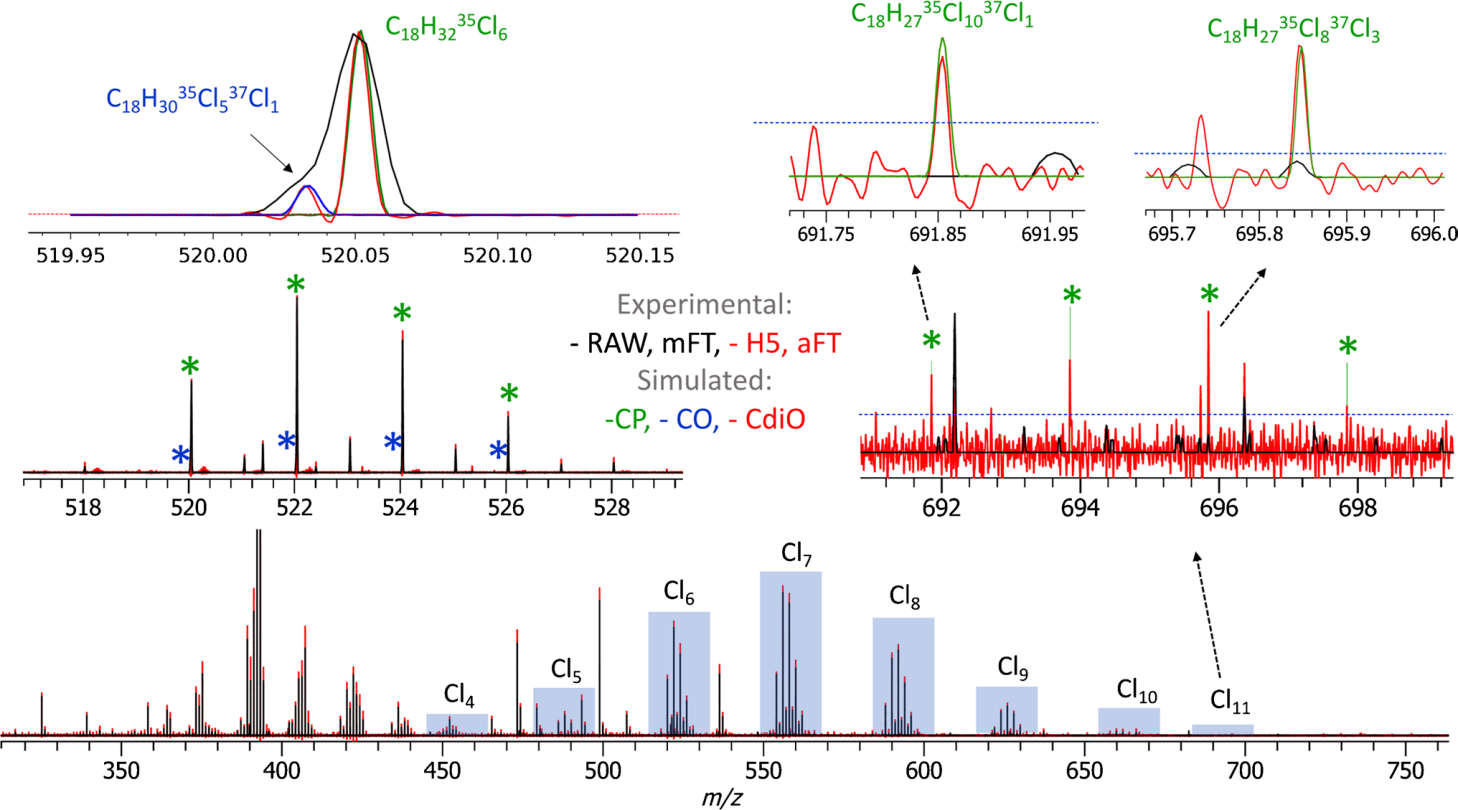
Polychlorinated compound analysis with an LTQ Orbitrap XL equipped with a glow discharge ion source and a high-performance DAQ/P system. Results of averaging of 100 transients are shown (negative ion polarity, resolution setting 30,000 at *m*/*z* 400, AGC 1e6, ITmax 200 ms). Mass spectra representation in the aFT mode (.H5, full profile, shown in red) resolves doublets that remain unresolved in the mFT mode (.RAW, reduced profile, shown in black) spectral representation. The identified compound classes are the chlorinated paraffins (CP, simulated peaks are shown in green) and chlorinated olefins (COs, simulated peaks are shown in blue), detected as nitrate anions, [M + NO_3_]^−^

Moreover, the averaged data unveils an expansion in the spectral dynamic range reaching five orders of magnitude (Figure S2, insets). This significant performance enhancement is attributed to the averaging of unreduced data, whether transients or full-profile aFT mass spectra, both of which yield comparable outcomes. When mFT mass spectra are averaged in full or reduced profile mode, the resultant increase in the spectral dynamic range is less stark [29, 48]. This distinction is further emphasized when comparing aFT and mFT mass spectra (Fig. 2, insets).

An analysis using the calibration mixture in the negative ion mode was carried out to assess further the developed experimental set-up’s aptitude for analyzing CP-containing samples. The results validate the high performance of the set-up in the negative ion mode, including an expected increase in resolution by a factor of 2 in the aFT mode when compared to the mFT mode and an overhead of circa 26 ms between the transients (data not shown). The overheads observed in the negative ion mode (26 ms) align with those noted in the positive ion mode (25 ms), and other performance improvements, shown in Fig. 1. In summary, the data derived from the calibration mixture indicate the platform’s potential suitability for CP analysis.

### Polychlorinated paraffin sample analysis: CP, CO, and CdiO compounds

Following the platform benchmarking described above, the LS-APGD Orbitrap FTMS equipped with the high-performance DAQ/P system was evaluated for the analysis of CP-containing samples. A single-chain C18-CP material of medium chlorination degree was analyzed using the ESI and LS-APGD LTQ Orbitrap XL FTMS and compared with data published earlier, using the APCI time-of-flight (TOF) MS [23]. The APCI TOF MS data revealed presence of seven CP homologs, from Cl_4_ to Cl_10_ (Table 1). The ESI LTQ Orbitrap XL FTMS analysis resulted in a similar CP-like distribution formed by the [M + Cl]^−^ adducts (data not shown). A detailed analysis of the CP-containing samples performed with the LS-APGD LTQ Orbitrap XL FTMS at *R* = 30,000 resolution setting is shown in Fig. 2.

**Table 1:** Elemental compositions and monoisotopic masses of neutral and charged C_18_-CP species with a general formula C_n_H_2n+2-x_Cl_x_, *n* = 18. The reference values are calculated using the FTMS data simulations (FTMS Simulator, Spectroswiss)

Chlorination degree	Molecule	M monoisotopic neutral, Da	M monoisotopic [M + Cl]^−^, *m*/*z*	M monoisotopic [M + NO_3_]^−^, *m*/*z*
4	C_18_H_34_Cl_4_	390.1414	425.1109	452.1298
5	C_18_H_33_Cl_5_	424.1024	459.0719	486.0908
6	C_18_H_32_Cl_6_	458.0635	493.0329	520.0519
7	C_18_H_31_Cl_7_	492.0245	526.9939	554.0129
8	C_18_H_30_Cl_8_	525.9856	560.9550	587.9739
9	C_18_H_29_Cl_9_	559.9466	594.9160	621.9350
10	C_18_H_28_Cl_10_	593.9076	628.8770	655.8960
11	C_18_H_27_Cl_11_	627.8686	662.8380	689.8570

The soft ionization conditions in the LS-APGD MS supported the preferential formation of nitrate-adduct [M + NO_3_]^−^ ions (Fig. 2 and Table 1). Therefore, the following study focused on the analysis of the [M + NO_3_]^−^ ions. The presence of nitric acid or nitrate salts has previously been reported to produce nitrate-adduct ions under ESI conditions [49]. As none of those was present in the sample, it is likely that the NO_3_^−^ can be generated either by electron ionization or by charge transfer from the ionized plasma gas [50–52]. The presence of the NO_3_^−^ compounds is supported by comparing the experimental and simulated data (Figure S3, Supporting Information).

The expected resolution benefits of the aFT mass spectra representation (visualized in Fig. 2, top left inset) are complemented by the increase in the S/N of the low-abundant components (visualized in Fig. 2, top right inset). Due to the data averaging of 100 scans, the abundance of the aFT peaks significantly exceeds that of the mFT ones. As a result, a series of CP containing from 4 to 11 chlorine atoms can be detected. For example, even the very low abundance Cl_11_ group of C_18_-CP components, such as C_18_H_27_^35^Cl_10_^37^Cl_1_ and C_18_H_27_^35^Cl_8_^37^Cl_3_, can be detected and assigned (Fig. 2, top right inset). Notably, it was not reported in the original study with APCI TOF MS [23]. Moreover, due to the increase in resolution and sensitivity, an additional class of compounds, the CO, can be now resolved. The CP and CO mass spectra strongly interfere, and it is crucial in the CP analysis to separate the two. Therefore, since new techniques in the CP analysis are now shifting to longer CP, the relevance of the identification of CO and CdiO is increasing. As an example, a CP compound C_18_H_32_^35^Cl_6_ is now distinguished from a CO compound C_18_H_30_^35^Cl_5_^37^Cl_1_ (Fig. 2, top left inset). The accurately simulated profiles of the peaks corresponding to CP and CO components are added to the mass spectra to facilitate the visualization of the results.

**Fig. 3:**
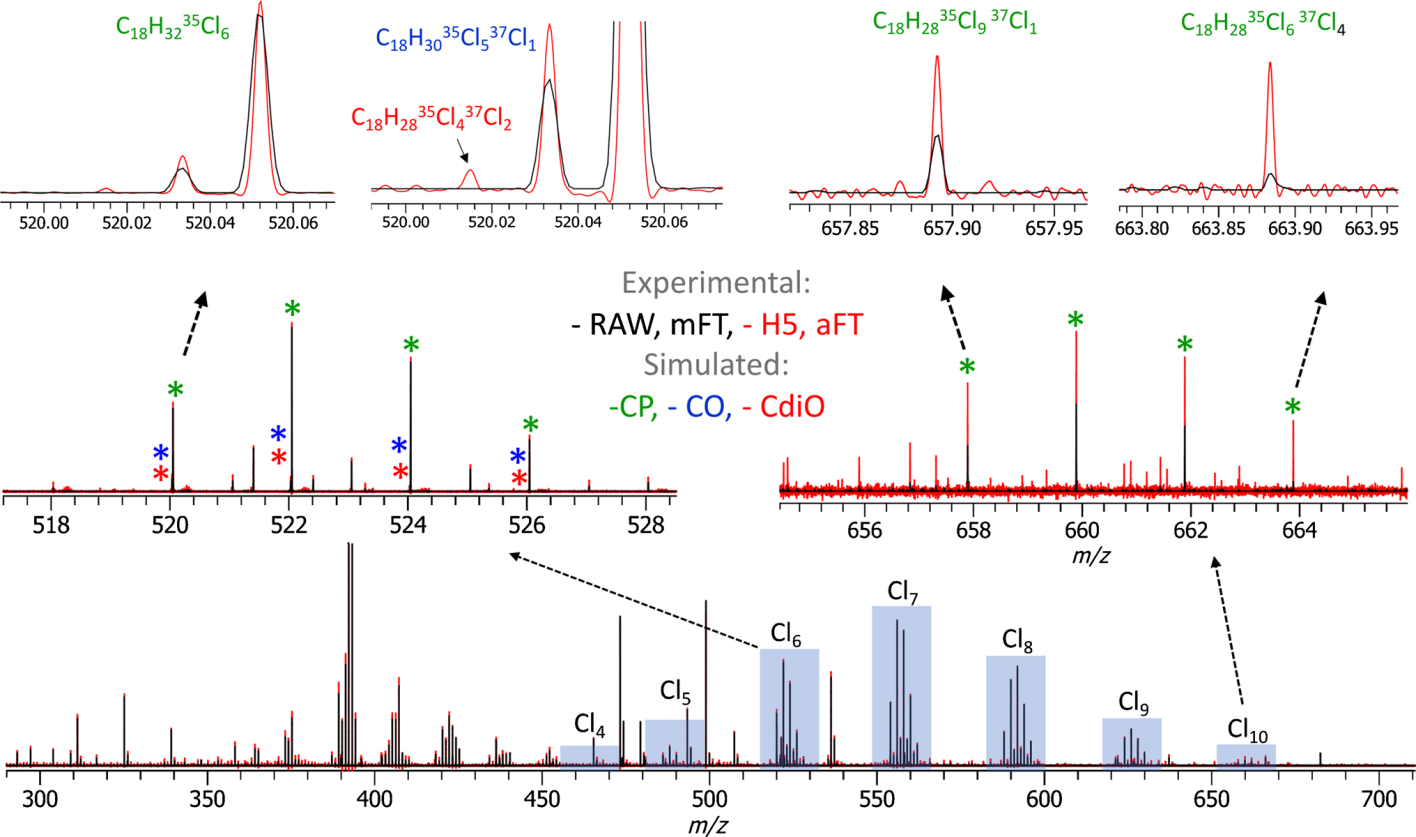
Polychlorinated compounds analysis with an LTQ Orbitrap XL equipped with a glow discharge ion source and a high-performance DAQ/P system. Results of averaging of 100 transients are shown (negative ion polarity, resolution setting 100,000 at *m*/*z* 400, AGC 1e6, ITmax 200 ms). Mass spectra in the aFT mode (.H5, full profile, shown in red) show identified triplets that remain unidentified in the mFT mode (.RAW, reduced profile, shown in black) spectral representation. The identified compound classes are the chlorinated paraffins (CP, shown in green), chlorinated olefins (COs, shown in blue), and chlorinated di-olefins (CdiOs, shown in red) detected as nitrate ions, [M + NO_3_]^−^

The increase to *R* = 100,000 at *m*/*z* 400 generated the original Orbitrap transient of 1536 ms and the in-hardware phased transient (H5) of ca. 1570 ms (an expected ~30 ms overhead), which translated into the corresponding increases in selectivity and sensitivity in comparison with the *R* = 30,000 measurements (Fig. 3). Similar to the results reported above (Fig. 2), data averaging of the unreduced aFT mass spectra (or transients) allowed to significantly enhance S/N values for the low-abundance compounds. For example, the detection of the Cl_10_ group of CP compounds C_18_H_28_^35^Cl_9_^37^Cl_1_ and C_18_H_28_^35^Cl_6_^37^Cl_4_ is shown in Fig. 3, top right inset.

Moreover, further increased resolution and sensitivity performance enabled the detection of another class of compounds—CdiO. Figure 3, top left inset, exemplifies the detection of the corresponding Cl_6_ group CdiO compound C_18_H_28_^35^Cl_4_^37^Cl_2_ in addition to the CP and CO compounds, C_18_H_32_^35^Cl_6_ and C_18_H_30_^35^Cl_5_^37^Cl_1_, respectively. The latter two compounds were identified at the 30,000 resolution setting (Fig. 2), but not the CdiO compound.

**Fig. 4:**
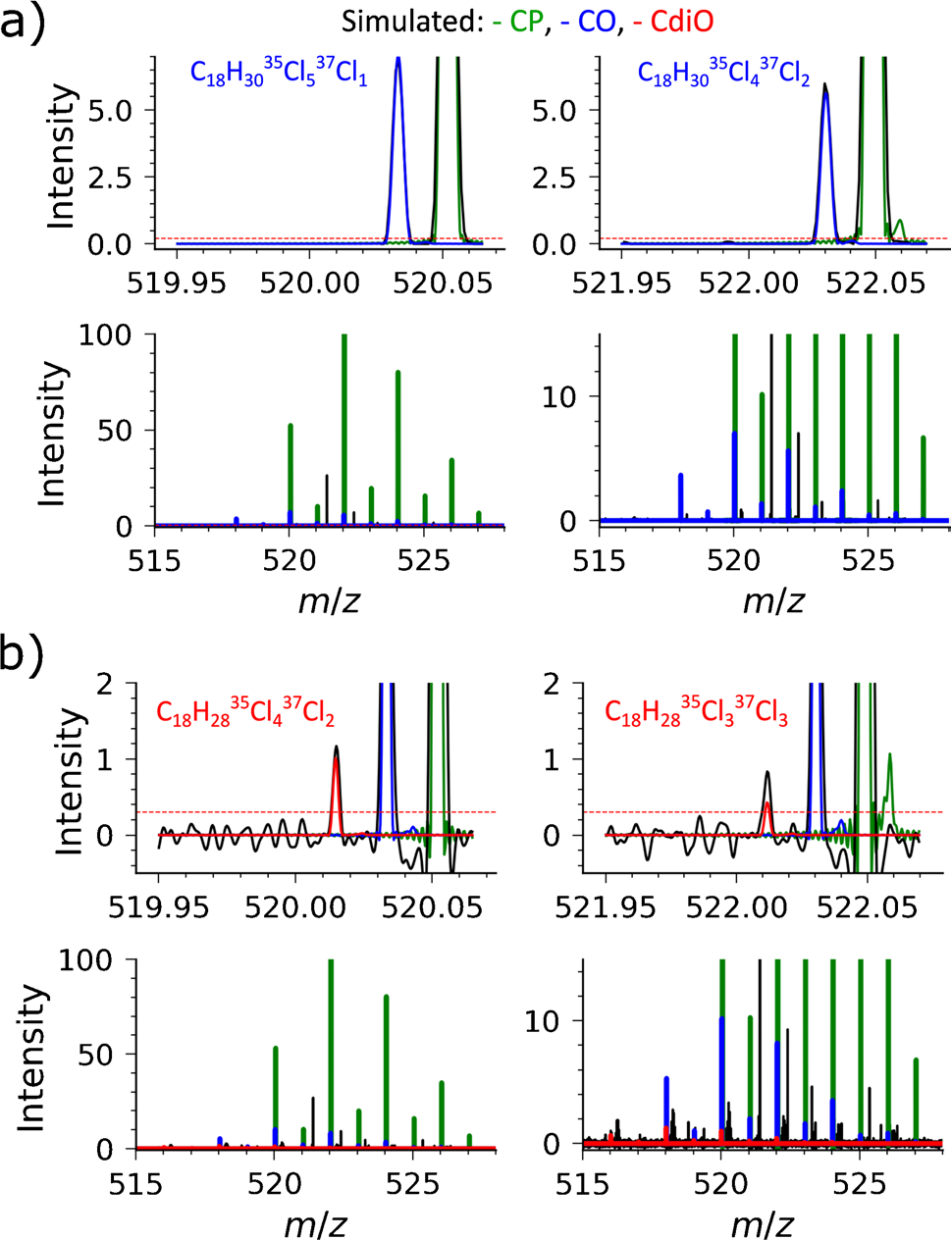
Identification of CP, CO, and CdiO components in the mass spectra as presented in Fig. 3. The Cl_6_ constituents of the CP and CO classes can be identified in the original mFT mass spectra (RAW) (**a**) and in the aFT mass spectra (H5). The corresponding CdiO compounds are detected only in the more sensitive mass spectra (H5, aFT) (**b**). Here, the experimental data are shown in black and simulated: CP—green, CO—blue, and CdiO—red. Experimental data and simulated isotopic envelopes normalized on the highest isotopologue

Figure 4 presents further evidence for the detection of all three classes—CP, CO, and CdiO—in the analysis of the CP-containing samples. The original mFT mass spectra (Fig. 4a) do not provide sufficient sensitivity and spectral dynamic range to detect the CdiO compounds, whereas the aFT mass spectra delivered by the experimental set-up in this work (Fig. 4b) enable their detection. The simulated peak profiles of the corresponding compounds are added to the experimental mass spectra to highlight the expected position and peak shape of the peaks of interest.

### Polychlorinated paraffin sample analysis: intensity error analysis

Accuracy and precision with which peak intensities are described in the mass spectra are essential for appropriate sample analysis, including quantitation and isotopic ratio analysis. Figures 2, 3, and 4 already imply that the aFT mass spectra provide higher intensities for the low-abundant components compared to the mFT data. We performed intensity error analysis for the mass spectra presented above to quantify these differences.

The intensity error analysis method is briefly described below. First, the elemental compositions of the CP were used as the entries for the accurate simulation of the corresponding peak profiles using the FTMS Simulator tool [30]. The thus obtained simulated data were compared with the experimental peak profiles (Figure S4, Supporting Information). Intensity errors were obtained for each isotopologue in the CP distribution, and isotopic-envelope-wide intensity errors were averaged to yield the final average intensity errors (Fig. S4c, f, and Tables S1 and S2, Supporting Information). The mass accuracy values reported in Tables S1 and S2 correspond to the original external mass scale calibration of the RAW mass spectra. The aFT mass spectra calibration was performed using the same calibration coefficients and equations, demonstrating comparable mass accuracies.

**Fig. 5:**
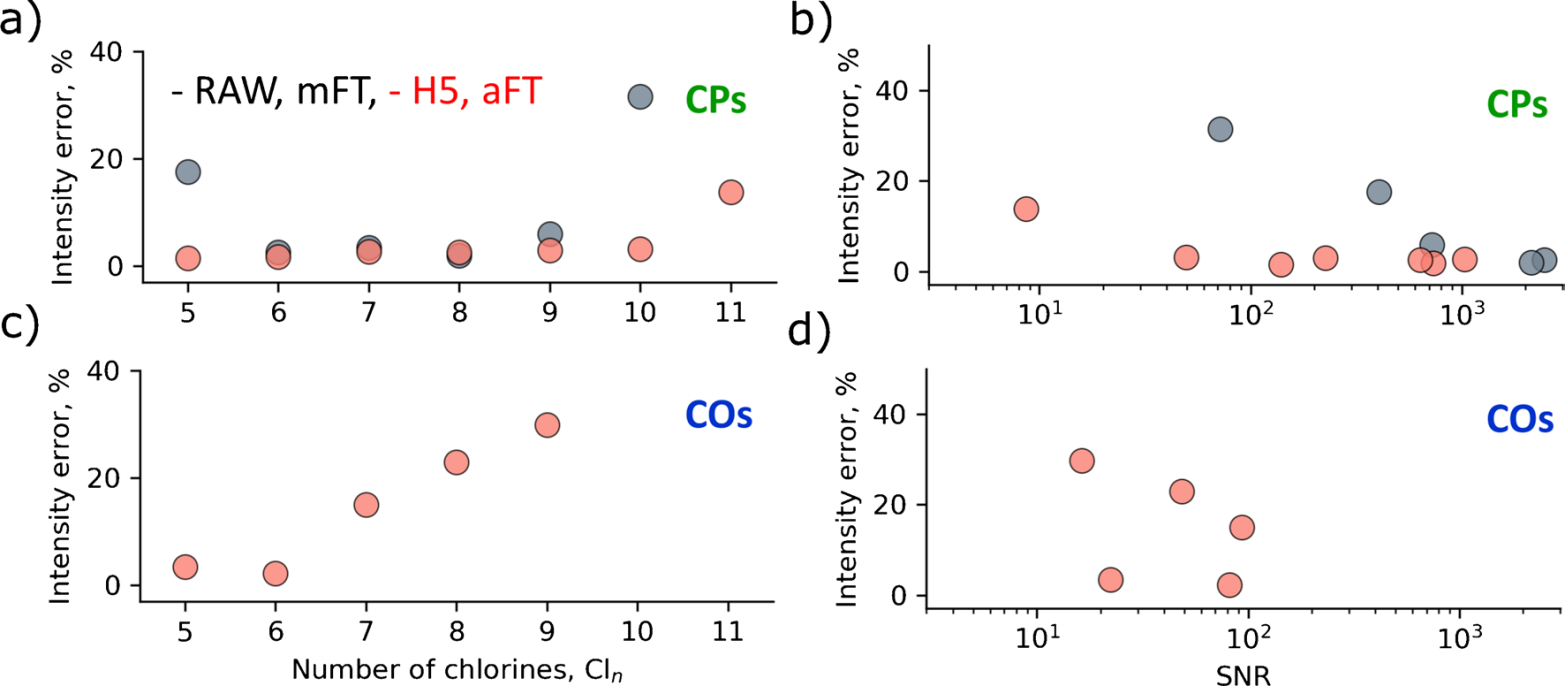
Intensity error distributions for the experimental data as shown in Fig. 2 (*R* = 30,000) as a function of the number of chlorines (**a**, **c**) and signal-to-noise ratio (S/N) (**b**, **d**). The intensity error calculation approach is discussed in Figure S4. **a**, **b** Results for the CP, and **c**, **d** for the CO

**Fig. 6:**
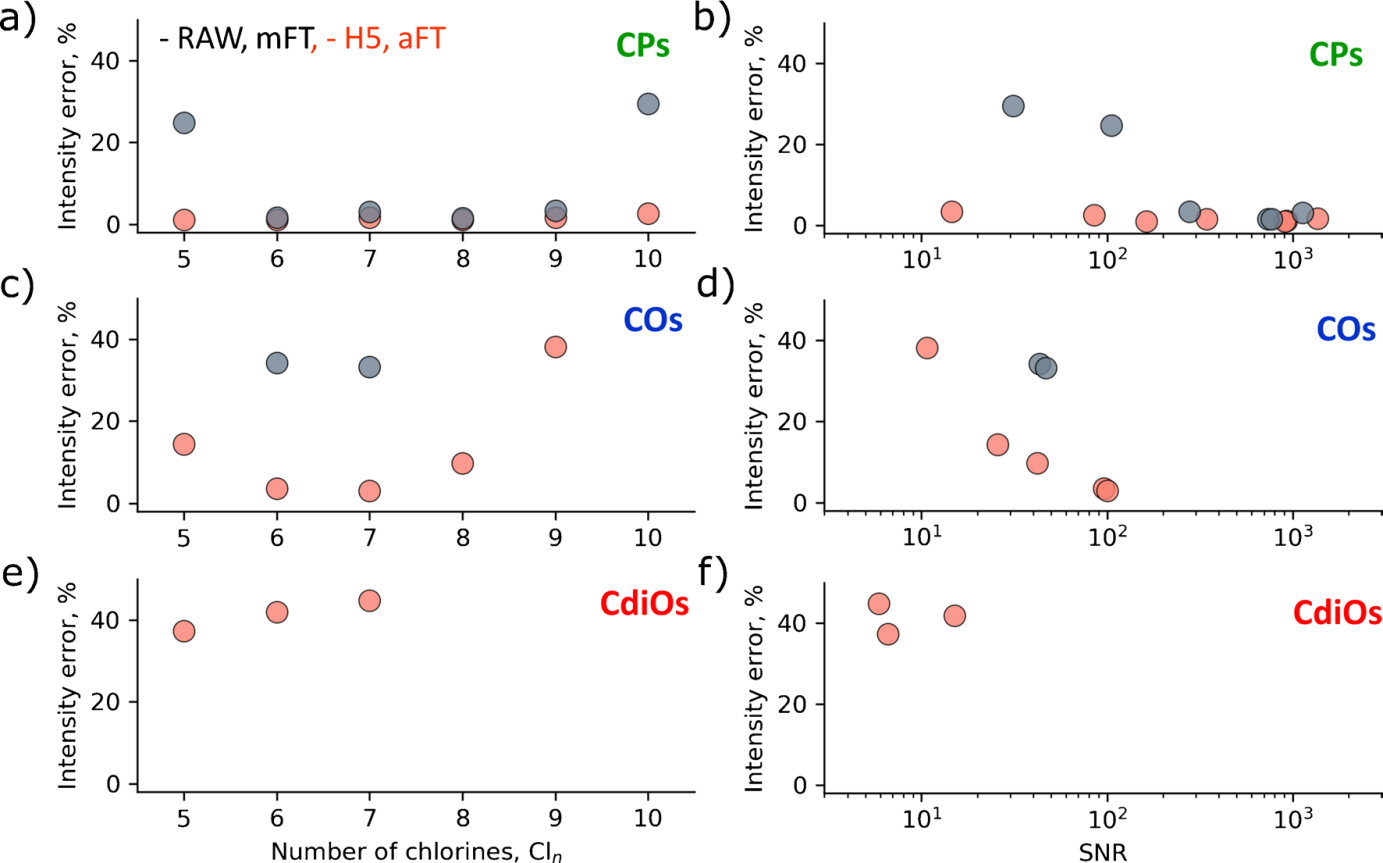
Intensity error distributions for the experimental data as shown in Fig. 3 (*R* = 100,000) as a function of the number of chlorines (**a**, **c**, **e**) and signal-to-noise ratio (S/N) (**b**, **d**, **f**). The intensity error calculation approach is discussed in Figure S4. **a**, **b** Results for the CP; **c**, **d** for the CO; and **e**, **f** for the CdiO

The intensity errors were thus calculated for all CP detected in Figs. 2 and 3 and plotted against the number of chlorines and S/N values for the 30,000 and 100,000 resolution cases (Figs. 5 and 6), correspondingly.

Mass spectra represented in both mFT (shown in gray) and aFT (shown in red) modes are compared. The aFT results demonstrate a significant reduction in intensity errors and a higher number of identified species, particularly for the low-abundant Cl_n_ groups. CP compounds by an order of magnitude dominate CO species, which, in turn, are about an order of magnitude more abundant than the CdiO species.

## Conclusions

Hyphenation between the LS-APGD ionization source and the LTQ Orbitrap XL mass spectrometer, connected in parallel to a high-performance DAQ/P system, enhanced the depth of CP complex mixture characterization. The versatility of LS-APGD, as a soft ionization technique, has been demonstrated. The LS-APGD showed an ability to generate CP-nitrate-adduct [M + NO_3_]^−^ ions. The unique feature of the DAQ/P hardware and firmware is its capacity for in-hardware-phased transient acquisition. This allowed for the aFT mass spectra representation even with the mFT-only foundational Orbitrap model. Equipped with this advanced aFT technology, our experimental set-up amplified resolution, sensitivity, and spectral dynamic range. This resulted in the successful resolution of interferences between CP, chlorinated olefins (COs), and chlorinated di-olefins (CdiOs)—three separate compound classes typically present in complex CP mixtures—at different degrees of chlorination. Furthermore, the aFT data demonstrated its superior performance to the original mFT data also in reporting the intensities of the mass spectral peaks, particularly the low abundant ones. Given that longer chains and increased chlorination degrees signify a surge in CP-related interferences, these findings and analytical capabilities hold significance for the evolution of analytical methods targeting analysis of complex mixtures in general and CP in particular. Further work should address analysis of CP with different numbers of carbon atoms and quantitation. The demonstrated capability of the developed system to enhance sensitivity and dynamic range by averaging results of multiple measurements (transients or aFT mass spectra) supports its further evaluation for the trace analysis.

## Supplementary Information

Below is the link to the electronic supplementary material.

Supplementary file1 (PDF 981 KB)
