# Supplementary material for: Evaluation of atmospheric-plasma-source absorption mode Fourier transform Orbitrap mass spectrometry for chlorinated paraffin mixtures
Source: Anal Bioanal Chem. 2024 Aug 13;416(23):5133–44. doi: 10.1007/s00216-024-05450-2 (PMC11377688; doi:10.1007/s00216-024-05450-2)
Supplement: Supplementary file 1 — Supplementary file1 (PDF 981 KB) [file 216_2024_5450_MOESM1_ESM.pdf]

Supporting Information.

Evaluation of Atmospheric-Plasma-Source Absorption Mode Fourier Transform Orbitrap Mass Spectrometry for Chlorinated Paraffin Mixtures

Claudia Masucci[1,2], Konstantin O. Nagornov[3], Anton N. Kozhinov[3], Kevin Kraft[1,2], Yury O. Tsybin[3], and Davide Bleiner[1,2,*]

[1] *Swiss Federal Laboratories for Materials Science and Technology, Überlandstrasse 129, 8600 Dübendorf, Switzerland*

[2] *Department of Chemistry, University of Zürich, Winterthurerstrasse 190, 8057 Zürich, Switzerland*

[3] *Spectroswiss, 1015 Lausanne, Switzerland*

* E-mail: davide.bleiner@empa.ch, Phone: +41 58 765 65 21. Fax: +41 58 765 69 63;

Table	Caption	Page
S1	Intensity error calculation for CP analysis: mFT spectrum	S-2
S2	Intensity error calculation for CP analysis: aFT spectrum	S-3

Figure	Caption	Page
S1	A schematic representation of the experimental set-up	S-4
S2	Calibration mixture analysis in a positive ion mode: 100 scans	S-5
S3	C18-CP analysis: experimental and simulated data comparison	S-6
S4	Intensity error analysis method	S-7

Table S1. Intensity error calculation approach application to CP analysis, as presented in Figure S4. The table presents results for a RAW (mFT) mass spectrum. The final intensity error is presented as an average value. The reference intensity values are calculated using accurate FTMS data simulations (FTMS Simulator, Spectroswiss).

Peak	*m/z*, ref.	Int., ref.	*m/z*, exp.	Int., exp.	ppm	Int. err, %
A	655.8960	99.8945				
A+1	656.8993	20.0260				
A+2	657.8931	320.2776	657.8917	132.8272	-2.132	-58.53
A+3	658.8964	64.1358				
A+4	659.8902	462.5049	659.8885	462.5049	-2.58	0
A+5	660.8935	92.5927				
A+6	661.8872	395.8203	661.8853	386.7893	-2.919	-2.28
A+7	662.8905	78.9849				
A+8	663.8843	222.2017	663.8819	76.62566	-3.599	-65.52
A+9	664.8876	44.3727				

Table S2. Intensity error calculation approach application to CP analysis, as presented in Figure S4. The table presents results for an H5 (aFT) mass spectrum. The final intensity error is presented as an average value. The reference intensity values are calculated using accurate FTMS data simulations (FTMS Simulator, Spectroswiss).

Peak	*m/z*, ref.	Int., ref.	*m/z*, exp.	Int., exp.	ppm	Int. err, %
A	655.8960	99.8945				
A+1	656.8993	20.0260				
A+2	657.8931	320.2776	657.8917	298.3327	-2.09	-6.85
A+3	658.8964	64.1358				
A+4	659.8902	462.5049	659.8887	462.5049	-2.276	0
A+5	660.8935	92.5927				
A+6	661.8872	395.8203	661.8856	430.7041	-2.471	8.81
A+7	662.8905	78.9849				
A+8	663.8843	222.2017	663.8829	223.4857	-2.185	0.58
A+9	664.8876	44.3727				

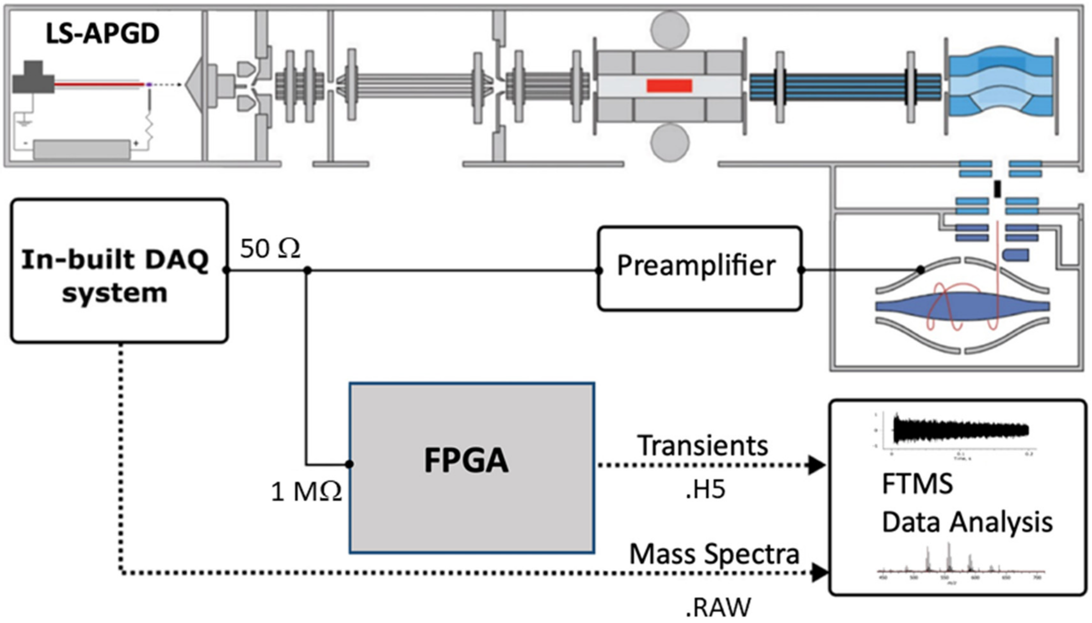

Figure S1. A schematic representation of the experimental set-up. The hybrid linear ion trap (LTQ) Orbitrap Fourier transform mass spectrometer (FTMS), LTQ Orbitrap XL™ (Thermo Fisher Scientific) is hyphenated with a liquid sampling – atmospheric pressure glow discharge (LS-APGD) plasma ion source. Ions are captured in the LTQ before their transfer into the Orbitrap mass analyzer via the C-trap. Ion signals are generated with the induced ion current detection system of the Orbitrap mass spectrometer. After the pre-amplifier, the differentially amplified ion signals as analog time-domain transients are sampled (digitized) with a conventional original manufacturer's in-built data acquisition system (DAQ) and yield mass spectra in the magnitude mode FT (.RAW mass spectra). In this work, we added an external high-performance DAQ/P system (FTMS Booster X2, Spectroswiss) to sample the time-domain ion signals at the output of the pre-amplifier in parallel to the in-built DAQ system. The outputs from both DAQ systems are processed with the dedicated data processing software, Peak-by-Peak (Spectroswiss).

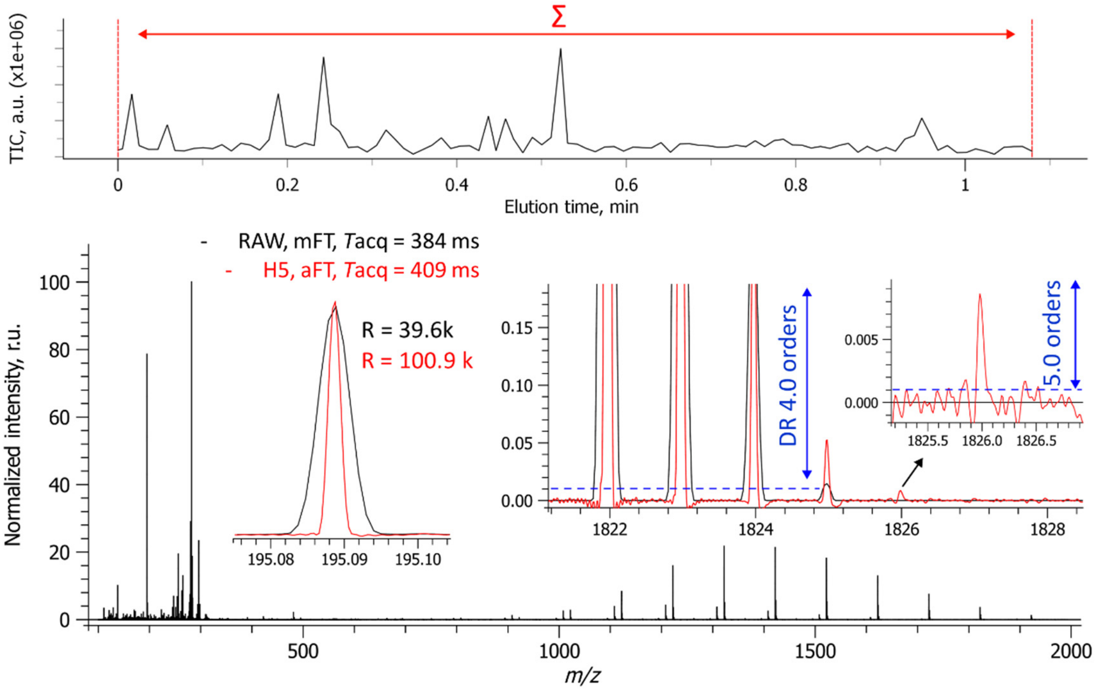

Figure S2. Calibration mixture analysis in a positive ion mode with an LTQ Orbitrap XL equipped with a glow discharge ion source and a high-performance DAQ/P system. Results of averaging of 100 transients are shown (resolution setting 30,000 at *m/z* 400, AGC 2e6, ITmax 200 ms). The original Orbitrap transient is at 384 ms, as expected, whereas the H5 transient is at 409 ms (25 ms overhead). The resolution increase between the mFT and aFT mass spectra confirms the high interscan frequency stability of ions. An excellent spectral dynamic range of 5 orders of magnitude is shown for averaged data.

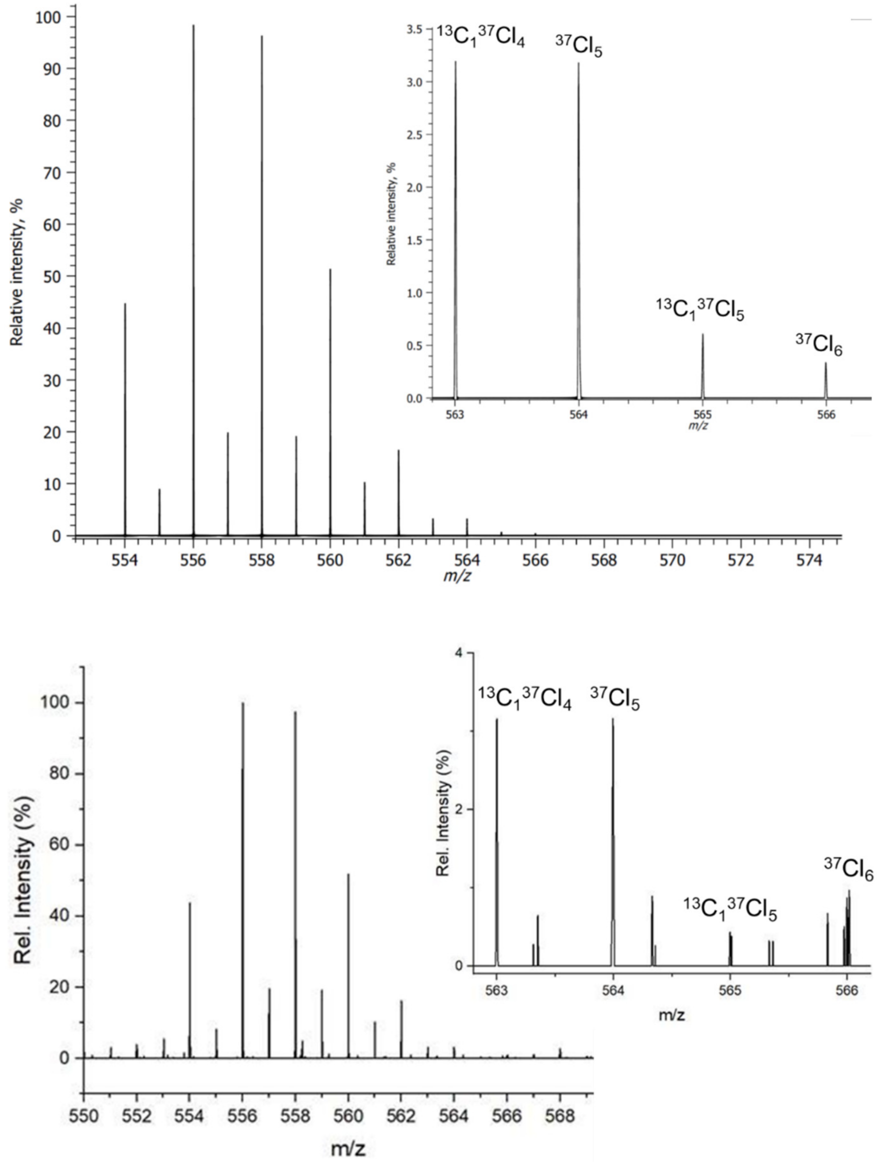

Figure S3. Chlorinated paraffins (C_{18}-CPs) analysis with an LTQ Orbitrap XL and aFT mass spectra representation. Shown is the selected C_{18}-CP cluster (chlorination degree x=7) formed with NO_3^- adducts, $[C_{18}H_{31}Cl_7+NO_3]^-$ radical anions. Experimental data (bottom panel) correlates with the simulated data (top panel) in a high spectral dynamic range. The simulated isotopic envelope was generated using FTMS Simulator (Spectroswiss).

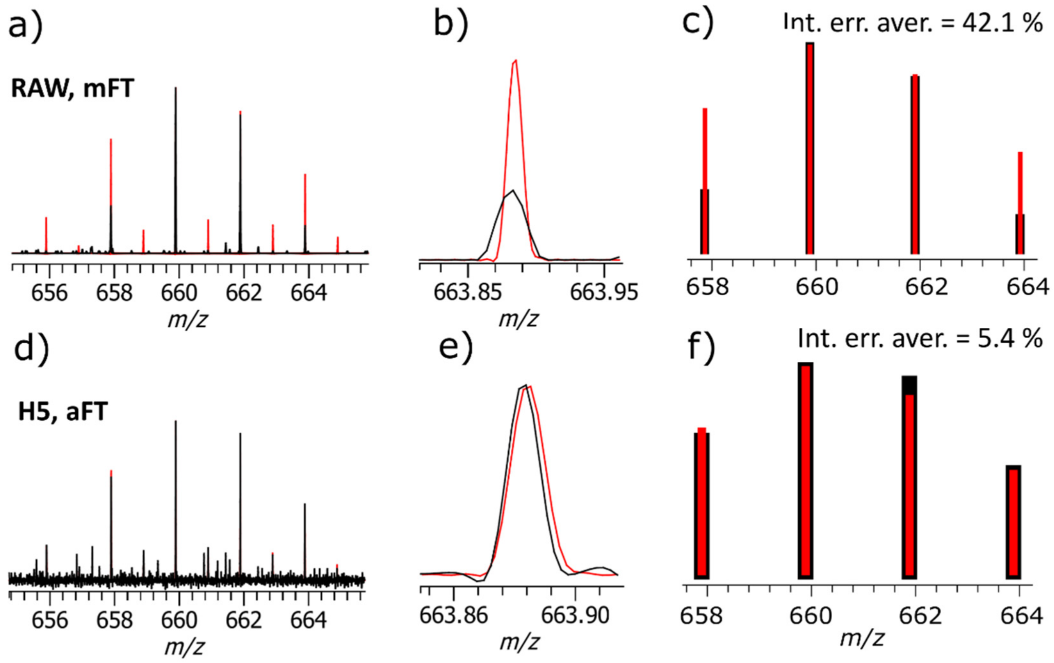

Figure S4. Intensity error analysis of data as shown in Figure 2. Intensity errors (averaged) were calculated based on the four highest isotopologues in each isotopic envelope, and experimental and simulated distributions were compared. Both experimental (black) and simulated (red) distributions were normalized on the highest isotopologue. Examples shown in panels (a) and (d) correspond to the $C_{18}H_{28}Cl_{10}$ (CP) compound with a NO_3^- ion and panels (b) and (e) - $C_{18}H_{28}{}^{35}Cl_6{}^{37}Cl_4$ isotope. Numerical values for the examples shown are detailed in Tables S1 and S2.
